# Providing psychiatric diagnosis and intervention in patients with chronic medical illness in the community: A novel collaboration between the Psychiatry team and the community team in a Singapore restructured hospital

**DOI:** 10.1192/j.eurpsy.2024.1253

**Published:** 2024-08-27

**Authors:** W. S. Lee, G. S. Lim, H. Y. Goh, Y. S. Chan

**Affiliations:** Psychiatry, Tan Tock Seng Hospital, Singapore, Singapore

## Abstract

**Introduction:**

Home nursing and medical services have an established role in delivering chronic medical care to populations which face difficulty accessing physical clinics. Those with chronic medical conditions and reduced mobility face a higher likelihood of suffering from psychiatric co-morbidity. However, till date there has been limited research done on home-based psychiatric care in this population.

Since 2021, the Psychiatry department of Tan Tock Seng Hospital (TTSH) has been collaborating with TTSH Community Health Team (CHT) to manage potential psychiatric issues in community patients.

These patients would be discussed in a weekly multidisciplinary setting. If indicated, home visit by both teams for home-based assessment and treatment would be arranged, allowing for detection and treatment of psychiatric illness.

**Objectives:**

To demonstrate that the collaboration between the psychiatry team and CHT leads to diagnosis and treatment of psychiatric illness in a population that might otherwise have been unable to access psychiatric services.

**Methods:**

We performed a retrospective study on all referrals from the CHT to the psychiatry team, within the 2-year period of August 2021 to August 2023. We collected demographic information, psychiatric history prior to referral, reason for referral, outcome of multidisciplinary discussion, and outcome of the home visits (including diagnoses made, and medications initiated).

**Results:**

A total of 92 patients were referred by the CHT to the psychiatry team. Most were elderly with multiple medical co-morbidities; of note, a history of stroke was present in 24 of the referred patients.

Common reasons for referral include suspected mental illness, risk assessment, and management of behavioural issues.

28 of the referred patients did not have a prior psychiatric history at the point of referral. Among these, home visits involving the psychiatric team were done for 16 patients. 11 (68%) of these home visits led to diagnosis of a new psychiatric illness. 9 of these patients were initiated on psychotropic medications in the home setting.

**Conclusions:**

A significant proportion of patients (68% of home visits without prior psychiatric diagnosis) were newly diagnosed with psychiatric illness, allowing early psychiatric intervention to be delivered. This was achieved in a population with a high prevalence of multiple medical comorbidity and barriers to clinic-based psychiatric evaluation and treatment.

We propose future comparative studies into how the collaboration between the psychiatric team and community health team can improve the quality of life and caregiver experience of patients with chronic medical problems, as well as how the service had improved the confidence of the community health team in identifying and managing patients with possible psychiatric issues.

**Disclosure of Interest:**

None Declared

